# Positive Predictive Value of the WHO Clinical and Immunologic Criteria to Predict Viral Load Failure among Adults on First, or Second-Line Antiretroviral Therapy in Kenya

**DOI:** 10.1371/journal.pone.0158881

**Published:** 2016-07-06

**Authors:** Anthony Waruru, Hellen Muttai, Lucy Ng’ang’a, Marta Ackers, Andrea Kim, Fredrick Miruka, Opiyo Erick, Julie Okonji, Tolbert Ayuaya, Sandra Schwarcz

**Affiliations:** 1 US Centers for Disease Control and Prevention (CDC), Nairobi, Kenya; 2 US Centers for Disease Control and Prevention (CDC), Atlanta, United States of America; 3 Kenya Medical Research Institute, Kisumu, Kenya; 4 San Francisco Department of Public Health, San Francisco, United States of America; 5 University of California San Francisco, San Francisco, United States of America; Boston University, UNITED STATES

## Abstract

Routine HIV viral load (VL) monitoring is the standard of care for persons receiving antiretroviral therapy (ART) in developed countries. Although the World Health Organization recommends annual VL monitoring of patients on ART, recognizing difficulties in conducting routine VL testing, the WHO continues to recommend targeted VL testing to confirm treatment failure for persons who meet selected immunologic and clinical criteria. Studies have measured positive predictive value (PPV), negative predictive value, sensitivity and specificity of these criteria among patients receiving first-line ART but not specifically among those on second-line or subsequent regimens. Between 2008 and 2011, adult ART patients in Nyanza, Kenya who met national clinical or immunologic criteria for treatment failure received targeted VL testing. We calculated PPV and 95% confidence intervals (CI) of these criteria to detect virologic treatment failure among patients receiving a) first-line ART, b) second/subsequent ART, and c) any regimen. Of 12,134 patient specimens tested, 2,874 (23.7%) were virologically confirmed as treatment failures. The PPV for 2,834 first-line ART patients who met either the clinical or immunologic criteria for treatment failure was 34.4% (95% CI 33.2–35.7), 33.1% (95% CI 24.7–42.3) for the 40 patients on second-line/subsequent regimens, and 33.4% (95% CI 33.1–35.6) for any ART. PPV, regardless of criteria, for first-line ART patients was lowest among patients over 44 years old and highest for patients aged 15 to 34 years. PPV of immunological and clinical criteria for correctly identifying treatment failure was similarly low for adult patients receiving either first-line or second-line/subsequent ART regimens. Our data confirm the inadequacy of clinical and immunologic criteria to correctly identify treatment failure and support the implementation of routine VL testing.

## Introduction

In sub-Saharan Africa, there are an estimated 22.1 million adults aged 15 years and above living with HIV and 1.4 million of these reside in Kenya, the country with the fourth highest number of infected persons worldwide [1]. According to service delivery data, the number of persons receiving antiretroviral therapy (ART) in Kenya has dramatically increased from 5,000 in 2003 to more than 500,000 at the end of 2011 [2]. In Nyanza, a region in Western Kenya bordering Lake Victoria, HIV prevalence among adults and adolescents aged 15–64 years in 2012 was 15.1%, the highest in the country [3].

A concern for persons on ART, particularly in resource-constrained areas, is the development of treatment failure followed by the change to more expensive regimens. In developed countries, identification of treatment failure is done through routine viral load (VL) testing. However, the costs, small number of laboratories with expertise in measuring VL, and difficulty with reliable transport of specimens, has greatly limited the use of routine VL testing in most resource-limited settings. Although the World Health Organization (WHO) recently adopted new recommendations for VL testing as the preferred routine method to monitor patients on ART, recognizing that this may not be feasible in all settings, the WHO continues to recommend the use of CD4 and clinical monitoring to diagnose treatment failure and VL testing to confirm failure in order avoid unnecessary changes in regimens [4]. The preference for routine VL monitoring over clinical and immunologic criteria to detect treatment failure comes from studies that have demonstrated low positive and negative predictive values of these criteria to detect failure [5–9]. However, none of these studies specifically evaluated the performance of these criteria among patients who were receiving second-line or subsequent ART regimens. It is possible that in resource-limited settings the absence of information on the predictive value of clinical and immunologic criteria for identifying treatment failure among patients who have changed regimens may lead to an overreliance on these criteria rather than support for routine VL monitoring.

In 2008, targeted VL testing was initiated in Nyanza region for patients on first-line or second-line ART regimens suspected to be failing treatment and meeting national immunological or clinical criteria for treatment failure adopted from WHO. We measured the positive predictive value (PPV) of immunological and clinical criteria for treatment failure among adult patients on first-line, second-line or other ART regimens. In June 2012, the Kenya national HIV treatment guidelines were updated, adding routine VL testing at 6 and 12 months after ART initiation and thereafter one VL test per year, aligning with the 2013 WHO treatment guidelines [4, 10]. However, at the time of writing this paper, routine VL monitoring had not yet been implemented. This analysis is therefore based on the 2008 national treatment guidance for targeted VL testing.

## Methods

### Study setting, population, and measures

Targeted VL testing was offered at 180 of the 600 health facilities in Nyanza that provided HIV care and treatment services as of the end of 2011. These facilities included dispensaries, health centers, sub-district hospitals, district hospitals, provincial, and referral hospitals. The study period was from September 2008 to December 2011.

The Kenya National AIDS and Sexually Transmitted Disease Control Program established criteria for ART failure based on the WHO clinical and immunologic criteria [11]. Adult patients who had been receiving ART for at least six months and had a new or recurrent WHO clinical stage 3 or 4 condition, new or recurrent papular pruritic eruptions, a decline in the CD4 cell count or percentage to baseline, a decline of more than 50% in the CD4 count or percentage, or patients who had received more than 12 months of ART and failed to demonstrate an increase greater than or equal to 50 CD4 cells/μL or had CD4 cell counts that remained under 100 cells/μL were eligible for VL testing to confirm the need to change regimens.

To ensure adherence to VL testing guidelines, standardized laboratory request forms that included the indications for VL testing and job aids were developed in 2008 and revised in 2009. Health workers were trained to recognize the clinical and immunologic criteria for targeted VL testing, complete the laboratory requisition form, and interpret the test results. Additionally, the mechanism to transport the specimens and receive results were developed prior to implementation of targeted VL testing. The laboratory requisition form included patient demographic information, facility where patient was receiving care, the indications for VL testing, current and past ART regimens, and CD4 test dates and results. Note that only the clinician documented indication for VL testing was used regardless of the CD4 test results included on the laboratory requisition. Specimens collected from patients with reported poor adherence two weeks prior to VL testing, active infections, including newly diagnosed tuberculosis or fever, were not tested since these conditions may increase or cause transient VL increase.

**Fig 1:**
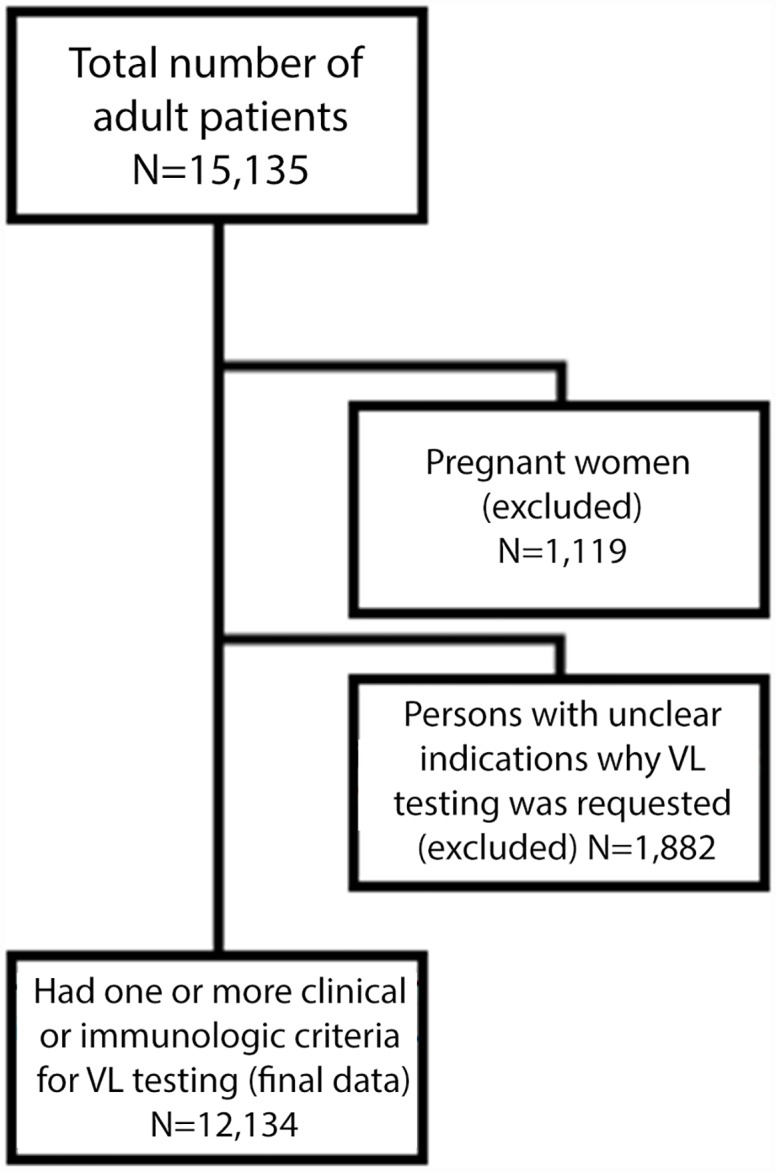
Inclusion of study subjects with clinical or immunological indication for viral load testing for analysis. This figure describes how the subjects were excluded from this analysis. We excluded 1,119 pregnant women and 1,882 persons with unclear indications why VL testing was requested.

This analysis includes patients aged 15 years and above for whom the indication for testing was documented on the requisition form. Because VL testing was used to routinely monitor ART virologic response among pregnant women who did not have clinical or immunologic criteria, we excluded this sub-population from the analysis (Fig 1). We used the CD4 test result that was closest to the date the VL specimen was obtained, provided that it occurred within 90 days prior to and not more than seven days after the VL specimen was obtained. Duration on ART was calculated from the date of the current ART regimen initiation to the date of specimen collection for all patients. Quantitative HIV RNA testing was conducted on plasma samples at the Kenya Medical Research Institute laboratory in Nyanza using the Roche COBAS Amplicor^™^ 1.5 [12].

### Statistical analysis

We calculated the PPV and corresponding 95% exact binomial confidence intervals of the clinical and immunologic criteria to identify virologic failure using Stata *diagt* command. Positive predictive value was calculated as the number of persons who met the clinical or immunologic criteria for viral load testing divided by the total number of persons with virologic failure Table 1. We defined failure using the current Kenyan and WHO definition of treatment failure (HIV RNA concentration equal to or above 1000 copies/mL) from a single specimen. We calculated separate PPVs for a) any of the criteria, b) any clinical criterion, and c) any immunologic criterion among patients receiving i) any regimen, ii) any first-line regimen, and iii) any second-line or other subsequent regimen. These analyses were categorized by age at VL testing, grouped into three age categories: aged 15–34 years, aged 35 to 44 years; and aged 45 years and older because of the relationship of age to health outcomes that might result in differences in the PPV within different age groups. All statistical analyses were conducted using Stata version 12.1 (Stata corporation, City, State) [13].

**Table 1:** Contingency table used for calculating positive predictive value.

	Virologic failure	
Clinical or immunologic criteria present	Yes	No
Yes	1940	3703
No	934	5557

PPV = 1,940/(1,940+3703) = 34.4%.

### Ethical considerations

Ethical approval for the study was obtained from the Kenya Medical Research Institute and United States Centers for Disease Control and Prevention.

## Results

A total of 12,134 adult patient records were included in the analysis. The majority of patients were receiving first-line ART; only 133, (1%) of patients were receiving second-line or subsequent regimens Table 2. The frequency distribution shows that there were more female patients (61.6%) than male, that patients aged 35–44 years accounted for the largest aged group (35.9%), and that most patients were seen at either a sub-district or district hospital (28.9% and 37.5%, respectively). There were a higher percentage of patients on second-line regimens aged 15 to 24 (6.0%) and aged over 54 years (13.5%) than those on first-line ART (3.8% and 11.5% respectively). Over half of the patients (6354) 52.4% had missing CD4 cell counts in the period 90 days prior to or more than one week after the VL specimen was obtained and a few (45/12,134) did not have CD4 value ever. Therefore, 6305 had CD4 values documented outside of this period (data not shown). Of the 5,780 patients for whom we had CD4 data, 17.8% had counts below 100 cells/ μL, 22.9% had cell counts between 100–199 cells/μL, 25.8% had counts between 200–350 cells/μL, 15.3% had counts between 351–500 cells/μL and 18.3% had counts above 500 cells/μL at the time of VL testing. Higher percentages of patients on second-line/other ART regimen had CD4 cell counts < 100 cells/μL and between 100–199 cells/μL than those on first-line treatment (18.8% and 15.8% vs 8.3% and 10.8%). Just over 10% of patients had been on therapy for one year or less and most, (78.8%) had never changed regimens. Over half of the patients on second-line therapy had been on ART for two years or less. Close to half of the patients met both clinical and immunologic criteria, while 5,076 (41.8%) patients were tested because of immunologic indications alone.

**Table 2:** Characteristics of adults who underwent targeted viral load testing, Nyanza, Kenya, 2008–2011.

Characteristic	Total	On first-line regimen*	On second-line^†^ or other^‡^ non-first-line regimen
	12134	12001	133
	n(%)	n(%)	n(%)
**Sex**			
Men	4658(38.4)	4603(38.4)	55(41.4)
Women	7476(61.6)	7398(61.6)	78(58.6)
**Age at VL testing (years)**			
15 to 24	459(3.8)	451(3.8)	8(6.0)
25 to 34	3153(26)	3118(26.0)	35(26.3)
35 to 44	4351(35.9)	4308(35.9)	43(32.3)
45 to 54	2777(22.9)	2748(22.9)	29(21.8)
55 to max	1394(11.5)	1376(11.5)	18(13.5)
**Year VL test was done**			
2008	94 (0.8)	93(0.8)	1(0.8)
2009	407(3.4)	402(3.3)	5(3.8)
2010	3354(27.6)	3313(27.6)	41(30.8)
2011	8279(68.2)	8193(68.3)	86(64.7)
**Facility**			
Dispensary/health center	2582(21.3)	2556(21.3)	26(19.5)
Sub-district hospital	3512(28.9)	3477(29.0)	35(26.3)
District hospital	4552(37.5)	4498(37.5)	54(40.6)
Provincial general hospital	84(0.7)	84(0.7)	0
Referral hospital	1404(11.6)	1386(11.5)	18(13.5)
**CD4 at VL request**			
< 100	1027(8.5)	1002(8.3)	25(18.8)
100 to 199	1322(10.9)	1301(10.8)	21(15.8)
200 to 350	1494(12.3)	1477(12.3)	17(12.8)
351 to 500	882(7.3)	881(7.3)	1(0.8)
> 500 to max	1055(8.7)	1052(8.8)	3(2.3)
Missing	6354(52.4)	6288(52.4)	66(49.6)
**Duration on ART (months)**			
6–12	1231(10.1)	1196(10.0)	35(26.3)
13–24	2751(22.7)	2714(22.6)	37(27.8)
25–36	2694(22.2)	2671(22.3)	23(17.3)
>37	4909(40.5)	4887(40.7)	22(16.5)
Missing	549(4.5)	533(4.4)	16(12)
**Regimen changes**			
Never changed	9559(78.8)	9559(79.7)	0
Ever changed	2575(21.2)	2442(20.3)	133(100)
**Indications for viral load testing**			
Clinical^§^ and immunologic^||^ criteria	6491(53.5)	6476(54.0)	15(11.3)
Clinical^§^ criteria only	567(4.7)	549(4.6)	18(13.5)
Immunological^||^ indications only	5076(41.8)	4976(41.5)	100(75.2)

*Includes nevirapine-based, efavirenz-based and two nucleotide-reverse transcriptase inhibitor regimens

^†^ Includes protease inhibitor and lopinavir-based regimens

^‡^ Includes other non-first line regimens that not in listed in the Kenya treatment guidelines

^§^ WHO stage 3 or 4 condition and/or new or recurrent papular pruritic eruptions and six months or more of antiretroviral therapy

^||^ Persistent CD4 cell count <100 cells/μL and more than 12 months of antiretroviral therapy, or CD4 cell count rise of <50 cells/μL and more than 12 months of antiretroviral therapy, or CD4 cell count rise of <50 cells/μL and more than 12 months of antiretroviral therapy, or CD4 cell count fall by >50% of peak and six months or more of antiretroviral therapy.

Of 12,134 patient specimens tested, 2,874 (23.7%) yielded an HIV RNA concentration > 1000 copies/mL, confirming virologic treatment failure. Among those with failure, the median RNA concentration was 41,150 copies/mL, interquartile range; 9,000–133,800. The PPV for virologic failure among all ART patients who met either the clinical or immunologic criteria for targeted VL testing was 34.4% (95% CI 33.1%-35.6%), 34.4% (95% CI 33.2%-35.7%) for first-line ART patients, and 33.1% (95% CI 24.7%-42.3% for patients who were on second-line or other non-first-line regimens Table 3. Among patients who met clinical but not immunologic criteria, the PPV for first-line ART patients was higher than the PPV for second-line ART patients,36.5% (95% CI 33.7%-39.4%) and 21.6% (95% CI 9.83%-38.2%), respectively. Among first-line ART patients, the PPVs dropped regardless of criteria category among the older age groups.

**Table 3:** Positive predictive value (PPV) of clinical and immunologic criteria for identifying treatment failure among HIV-infected adults receiving first-line or second-line/other antiretroviral therapy, Nyanza Province, 2008–2011.

Characteristics	Number of patients with virologic failure*	Clinical or immunologic indications	Clinical indications only	Immunologic indications only
	n(%)	PPV% (95% CI)	PPV% (95% CI)	PPV% (95% CI)
**Any regimen (N = 12134)**				
**Total**	**2874(23.7)**	**34.4(33.1–35.6)**	**36.0(33.3–38.8)**	**35.5(34.2–36.8)**
Age at viral load testing (years)				
15 to 34	1134(31.4)	45.5(43.1–47.9)	44.5(39.5–49.7)	47.2(44.7–49.8)
35 to 44	995(22.9)	33.6(31.5–35.8)	35.0(30.4–39.8)	34.8(32.6–37.1)
45 to oldest	745(17.9)	25.4(23.5–27.4)	28.3(23.7–33.2)	26.0(23.9–28.1)
**Patients receiving first-line antiretroviral therapy (n = 12001)**				
**Total**	**2834(23.6)**	**34.4(33.2–35.7)**	**36.5(33.7–39.4)**	**35.5(34.2–36.8)**
Age at viral load testing (years)				
15 to 34	1118(31.3)	45.6(43.2–48.0)	45.6(40.5–50.8)	47.3(44.7–49.8)
35 to 44	984(22.8)	33.7(31.6–35.9)	35.0(30.4–39.9	35.0(32.7–37.3)
45 to oldest	732(17.8)	25.3(23.3–27.3)	28.4(23.8–33.4)	25.9(23.8–28.0)
**Patients on second-line/other non-first-line ART (n = 133)**				
**Total**	**40(30.1)**	**33.1(24.7–42.3)**	**21.6(9.83–38.2)**	**35.0(25.7–45.2)**
Age at viral load testing (years)				
15 to 34	16(37.2)	41.0(25.6–57.9)	0.0(0.0–33.6)	45.7(28.8–63.4)
35 to 44	11(25.6)	27.5(14.6–43.9)	33.3(9.9–65.1)	27.3(13.3–45.5)
45 to oldest	13(27.7)	30.8(17.0–47.6)	25.0(7.3–52.4)	31.3(16.1–50.0)

*Viral load ≥1000 copies/mL.

## Discussion

Less than a quarter of the over 12,000 patient specimens meeting clinical or immunologic criteria for suspected treatment failure were confirmed to be failing treatment. The PPVs were equally low for patients on second-line/other non-first-line ART compared to patients receiving first-line ART, which likely reflects the lack of specificity of clinical signs and symptoms. Our findings are similar to those of other studies that have demonstrated poor PPV of either clinical or immunologic criteria for ART failure but now extend the findings to include patients receiving non-first-line regimens [5–7, 14–18]. As such, our findings provide additional evidence of poor performance of the criteria to detect treatment failure. Indeed our results indicate that if clinicians would have relied exclusively on the 2008 Kenya immunologic and clinical criteria and classified these patients as treatment failures, over 75% of patients would have been switched to new, more expensive, ART regimens unnecessarily.

Available evidence cannot support the use of clinical or immunologic criteria to accurately identify virologic failure. Presently, there is no other, more effective strategy for assessing treatment failure among ART patients than measuring their plasma viral levels [19]. Studies have demonstrated that dried blood spots (DBS) can be used to reliably and accurately quantify HIV RNA concentrations for patients with high levels of viremia, but their performance at identifying patients with lower VLs (e.g. between 1000–3000 copies/mL) is sub-optimal [20–22]. However, realizing this caveat of misclassification, using DBS as a source of specimens for monitoring VLs in settings without other options for quantifying viral levels appears feasible in many resource-constrained areas. A recent study compared VL measurements in adults and children obtained from plasma and capillary blood DBS in Nyanza and Nairobi regions of Kenya and found sensitivities greater than 87% and specificities greater than 94% for VLs of 1000 copies/mL or greater using DBS [23]. Applying these findings, in our study, DBS would have optimally and correctly identified virologic treatment failure among 2,564 (89.2%) of patient specimens who had VL greater than 3000 copies/mL.

A major strength of our data is that they originate from routine HIV care facilities and reflect clinicians’ suspicions of patients with treatment failure. Our data provide additional evidence for the poor performance of clinical and immunologic criteria to detect treatment failure regardless of the ART regimen, but there are limitations to consider. Because our specimens came from patients who were receiving routine HIV care and met at least one of the criteria for targeted VL testing, we were unable to calculate the negative predictive value. In addition to our study being limited by the patient selection, the data came from one region in Kenya and may not be representative of other areas of the country or elsewhere. Furthermore, the information regarding patients came exclusively from laboratory requisition forms and is limited in scope. Over half of the patients did not have CD4 data due to the asynchronous collection of CD4 and VL samples. Since the guidelines state that patients with poor adherence two weeks prior to VL testing, active infections, fever, or newly diagnosed with tuberculosis should not be tested for virologic failure, we were not able to verify if patients with some of these criteria were indeed failing treatment. The number of specimens from patients receiving non-first-line ART was small and resulted in less precise estimates that those obtained for patients on first-line ART; larger samples may have produced different results.

Our results indicate that the 2008 Kenya immunologic and clinical criteria may have misclassified too many persons as cases of suspected treatment failure and support the incorporation of routine VL testing as a component of ART monitoring in the Kenyan national treatment guidelines so as to prevent expensive unnecessary ART regimen changes. Younger patients had a higher proportion of treatment failure. It is important to closely monitor this dynamic population since they have more years of ART ahead of them. We also found that the sensitivity was lower for older persons who are at higher risk for a number of other, non-HIV-related conditions whose symptoms may be misclassified as indicators for viral load testing relative to younger persons. While we did find that the PPVs were slightly better for first-line regimens, they were still too poor to recommend continued use of targeted VL testing. As Kenya implements new guidelines on routine VL testing, careful attention will be needed to ensure that health workers are appropriately trained and processes developed for tracking samples and updating results in patient charts. It will be important to establish close monitoring and assess the impact of this change in clinical management. Routine VL data will eventually be useful addition to HIV case-based surveillance.

## Supporting Information

S1 Data Data used in the analysis. This file contains Stata data used for these analyses.
(DTA)

## References

[1] Joint United Nations Programme on HIV/AIDS (UNAIDS). UNAIDS report on the global AIDS epidemic 2013. 2013.

[2] Office of U.S. Global AIDS Coordinator and the Bureau of Public Affairs USSD. Annual Reports to Congress on the President's Emergency Plan for AIDS Relief. Available from: http://www.pepfar.gov/reports/progress/index.htm.

[3] National AIDS and STI Control Programme (NASCOP) K. Kenya AIDS Indicator Survey 2012: Final Report. Nairobi, Kenya2014.

[4] World Health Organization. Consolidated guidelines on the use of antiretroviral drugs for treating and preventing HIV infection: recommendations for a public health approach. Geneva, Switzerland 2013. 24716260

[5] Chaiwarith R, Wachirakaphan C, Kotarathititum W, Praparatanaphan J, Sirisanthana T, Supparatpinyo K. Sensitivity and specificity of using CD4+ measurement and clinical evaluation to determine antiretroviral treatment failure in Thailand. Int J Infect Dis. 2007 9;11(5):413–6. . Epub 2007/03/03. eng. 17331776 10.1016/j.ijid.2006.11.003

[6] Mee P, Fielding KL, Charalambous S, Churchyard GJ, Grant AD. Evaluation of the WHO criteria for antiretroviral treatment failure among adults in South Africa. AIDS. 2008 10 1;22(15):1971–7. Epub 2008/09/12. eng. 10.1097/QAD.0b013e32830e4cd8 18784460

[7] Meya D, Spacek LA, Tibenderana H, John L, Namugga I, Magero S, et al Development and evaluation of a clinical algorithm to monitor patients on antiretrovirals in resource-limited settings using adherence, clinical and CD4 cell count criteria. J Int AIDS Soc. 2009;12:3 . Pubmed Central PMCID: 2664320. Epub 2009/03/06. eng. 19261189 10.1186/1758-2652-12-3 PMC2664320

[8] Rewari BB, Bachani D, Rajasekaran S, Deshpande A, Chan PL, Srikantiah P. Evaluating patients for second-line antiretroviral therapy in India: the role of targeted viral load testing. J Acquir Immune Defic Syndr. 2010 12 15;55(5):610–4. Epub 2010/10/05. eng. 10.1097/QAI.0b013e3181f43a31 20890211

[9] van Oosterhout JJ, Brown L, Weigel R, Kumwenda JJ, Mzinganjira D, Saukila N, et al Diagnosis of antiretroviral therapy failure in Malawi: poor performance of clinical and immunological WHO criteria. Trop Med Int Health. 2009 8;14(8):856–61. Epub 2009/06/26. eng. 10.1111/j.1365-3156.2009.02309.x 19552661

[10] Ministry of Health; National AIDS and STI Control Program (NASCOP). Guidelines on use of antiretroviral drugs for treating and preventing HIV infection: a rapid advice, 2014. Nairobi, Kenya 2014.

[11] National AIDS/STI Control Program. Guidelines for Antiretroviral Therapy in Kenya. 4th Edition: 2011. Print. Nairobi, Kenya: National AIDS/STI Control Program; 2011.

[12] Roche Molecular Systems Inc. Roche Molecular Diagnostics. 1996–2013.

[13] Stata Coorporation. Stata. 12.1 ed Texas USA 1985–2011.

[14] Rutherford GW, Anglemyer A, Easterbrook PJ, Horvath T, Victoria M, Penazzato M, et al Predicting treatment failure in adults and children on antiretroviral therapy: a systematic review of the performance characteristics of the 2010 WHO immunologic and clinical criteria for virologic failure. 7th International AIDS Society (IAS) Conference on HIV Pathogenesis, Treatment and Prevention; Kuala Lumpur, Malysia2013. 10.1097/QAD.0000000000000236 24849476

[15] Keiser O, MacPhail P, Boulle A, Wood R, Schechter M, Dabis F, et al Accuracy of WHO CD4 cell count criteria for virological failure of antiretroviral therapy. Trop Med Int Health. 2009 10;14(10):1220–5. Pubmed Central PMCID: 3640048. eng. 10.1111/j.1365-3156.2009.02338.x 19624478 PMC3722497

[16] Moore DM, Awor A, Downing R, Kaplan J, Montaner JS, Hancock J, et al CD4+ T-cell count monitoring does not accurately identify HIV-infected adults with virologic failure receiving antiretroviral therapy. J Acquir Immune Defic Syndr. 2008 12 15;49(5):477–84. Epub 2008/11/08. eng. 10.1097/QAI.0b013e318186eb18 18989232

[17] Rawizza HE, Chaplin B, Meloni ST, Eisen G, Rao T, Sankale JL, et al Immunologic criteria are poor predictors of virologic outcome: implications for HIV treatment monitoring in resource-limited settings. Clin Infect Dis. 2011 12;53(12):1283–90. Pubmed Central PMCID: 3246873. Epub 2011/11/15. eng. 10.1093/cid/cir729 22080121 PMC3246873

[18] Reynolds SJ, Sendagire H, Newell K, Castelnuovo B, Nankya I, Kamya M, et al Virologic versus immunologic monitoring and the rate of accumulated genotypic resistance to first-line antiretroviral drugs in Uganda. BMC Infect Dis. 2012;12:381 Pubmed Central PMCID: 3548731. Epub 2012/12/29. eng. 10.1186/1471-2334-12-381 23270482 PMC3548731

[19] Laurent C, Kouanfack C, Laborde-Balen G, Aghokeng AF, Mbougua JB, Boyer S, et al Monitoring of HIV viral loads, CD4 cell counts, and clinical assessments versus clinical monitoring alone for antiretroviral therapy in rural district hospitals in Cameroon (Stratall ANRS 12110/ESTHER): a randomised non-inferiority trial. Lancet Infect Dis. 2011 11;11(11):825–33. Epub 2011/08/13. eng. 10.1016/S1473-3099(11)70168-2 21831714

[20] Fiscus SA, Brambilla D, Grosso L, Schock J, Cronin M. Quantitation of human immunodeficiency virus type 1 RNA in plasma by using blood dried on filter paper. Journal of Clinical Microbiology. 1998 1;36(1):258–60. . eng. 9431960 10.1128/jcm.36.1.258-260.1998 PMC124847

[21] Garrido C, Zahonero N, Corral A, Arredondo M, Soriano V, de Mendoza C. Correlation between Human Immunodeficiency Virus Type 1 (HIV-1) RNA measurements obtained with dried blood spots and those obtained with plasma by use of nuclisens EasyQ HIV-1 and Abbott RealTime HIV load tests. Journal of Clinical Microbiology. 2009 4;47(4):1031–6. Pubmed Central PMCID: 124847. eng. 10.1128/JCM.02099-08 19193847 PMC2668340

[22] Johannessen A, Garrido C, Zahonero N, Sandvik L, Naman E, Kivuyo SL, et al Dried blood spots perform well in viral load monitoring of patients who receive antiretroviral treatment in rural Tanzania. Clin Infect Dis. 2009 9 15;49(6):976–81. Epub 2009/08/12. eng. 10.1086/605502 19663598

[23] Schmitz ME, Agolory S, Umuro M, Junghae M, Ombayo J, Broyles LN, et al Performance of Dried Blood Spots prepared under clinical conditions to identify virologic failure among adults and children on antiretroviral therapy in Kenya. International AIDS Society Conference; Melbourne, Australia2014.

